# Linking Personality to Larval Energy Reserves in Rainbow Trout (*Oncorhynchus mykiss*)

**DOI:** 10.1371/journal.pone.0049247

**Published:** 2012-11-14

**Authors:** Madelene Åberg Andersson, Erik Höglund

**Affiliations:** DTU Aqua, Section for Aquaculture, The North Sea Research Centre, Technical University of Denmark, Hirtshals, Denmark; Cajal Institute, Consejo Superior de Investigaciones Científicas, Spain

## Abstract

There is a surging interest in the evolution, ecology and physiology of personality differences. However, most of the studies in this research area have been performed in adult animals. Trait variations expressed early in development and how they are related to the ontogeny of an animal’s personality are far less studied. Genetic differences as well as environmental factors causing functional variability of the central serotonergic system have been related to personality differences in vertebrates, including humans. Such gene-environment interplay suggests that the central serotonergic system plays an important role in the ontogeny of personality traits. In salmonid fishes, the timing of emergence from spawning nests is related to energy reserves, aggression, and social dominance. However, it is currently unknown how the size of the yolk reserve is reflected on aggression and dominance, or if these traits are linked to differences in serotonergic transmission in newly emerged larvae. In this study we investigated the relationship between yolk reserves, social dominance, and serotonergic transmission in newly emerged rainbow trout (*Oncorhynchus mykiss)* larvae. This was conducted by allowing larvae with the same emergence time, but with different yolk sizes, to interact in pairs for 24 h. The results show that individuals with larger yolks performed more aggressive acts, resulting in a suppression of aggression in individuals with smaller yolks. A higher brain serotonergic activity confirmed subordination in larvae with small yolks. The relationship between social dominance and yolk size was present in siblings, demonstrating a link between interfamily variation in energy reserves and aggression, and suggests that larger yolk reserves fuel a more aggressive personality during the initial territorial establishment in salmonid fishes. Furthermore, socially naïve larvae with big yolks had lower serotonin levels, suggesting that other factors than the social environment causes variation in serotonergic transmission, underlying individual variation in aggressive behavior.

## Introduction

Over recent decades, there has been a rising scientific interest in the causes and consequences of individual variation in behavior and physiological traits. This is reflected in the substantial number of reviews published on this topic [1]–[21], and the wealth of studies demonstrating that individual trait variability often shows consistency over time and between contexts (for references see review by Stamp and Grootius [22]). The terms personalities, behavioral syndromes, temperament and stress coping styles have been used to describe this constancy in individual suites of traits [10]–[13], [23], [24]. The majority of studies regarding animal personality have been performed in adult animals, and recently questions have been raised regarding the existence of such trait correlations early in development and the relation to personality traits expressed later in life [11], [22], [25].

In many animal groups mortality is most pronounced early in ontogeny, implying that selection would have the greatest impact on personality during this time of development [11]. In addition, there are studies demonstrating a certain degree of plasticity related to the individual response pattern, suggesting that experience plays an important role in the shaping of an animal’s personality. For example, in salmonid fishes previous social experiences have been shown to affect the behavioral response to an unfamiliar object [26], as well as the aggressive behavior performed during territorial defense [27]. Furthermore, social and environmental experiences early in ontogeny have been demonstrated to have profound long lasting effects on behavioral and physiological traits in primates and rodents [28], [29]. Even if the above studies indicate that the effects of selection and experience have a strong effect on individuals pronounced early in ontogeny, there is little information regarding presence of personalities during the early developmental stages and how they are reflected in traits expressed later in ontogeny.

Behavioral traits affected by early experiences may be reflected in long lasting alterations in brain function. For example, social isolation in the time window between post-weaning and adolescence has been demonstrated to have a persistent effect on behavior, brain architecture and neurotransmission in rats [29], [31]. Some of these changes are associated with alterations in the release and activity of serotonin (5-HT); a neurotransmitter/modulator with evolutionarily well-conserved functions [32], [34]. A relationship between 5-HT and personality is further demonstrated in male lizards (Anolis carolinensis) where males that take shorter time to mount a female and have a shorter response time to a food source when exposed to a new environment, also show a tendency to become socially dominant. This propensity for social dominance was reflected in a lower 5-HT-ergic brain activity prior to social interactions [35]. The latter study displays results that are consistent with the general consensus, that variability in the central 5-HT function is associated with personality differences, where low levels of this neurotransmitter are coupled with high levels of aggression [12]. Furthermore, 5-HT transmission is highly effected by the social environment, although most studies examining the relationship between aggressive behavior and 5-HT levels have been performed on socially experienced individuals (reviewed by De Boer [36]). Studies using socially naive individuals predisposed to different levels of aggression are needed to clarify the causative effects of 5-HT on aggression and other related behaviors.

In salmonid fishes, the timing of emergence is a crucial niche shift where larvae emerge from the spawning nest in search of exogenous feed and to establish a territory [33], [37], [38]. There are indications that individual variation in the timing of this shift is related to behavioral traits expressed by the individual. Metcalfe et al. [39] demonstrated that early emerging individuals were more aggressive and had a higher probability of becoming social dominant compared to late emerging individuals. Furthermore, variation in the amount of yolk reserves between early and late emerging larvae [30], [39] suggests a coupling between energy reserves and personality differences. A similar relation between energy and personality were recently demonstrated in offspring originating from selected rainbow trout strains exhibiting differences in boldness and propensity to become socially dominant. Females originating from the more dominant and aggressive strain produced larger eggs and larvae with larger yolk reserves compared to females originating from the less dominant and shyer strain [40]. In the latter study it was suggested that such differences in energy reserves, if persistent after emergence, could supply the energetic requirements for an aggressive behavior during territory establishment and defense. If a relationship between energy reserves and levels of aggression exists in newly emerged larvae it could predict the behavioral responses of an individual before the initial territorial defense. Moreover, if present, a relationship between such differences in socially naive individuals and 5-HT signaling could reveal if inherited differences underlie the shaping of an individuals’ personality.

Thus, the aims of the present study were to investigate whether the propensity to become dominant co-varied with the amount of energy reserves (yolk reserves), and how these factors are reflected in 5-HT signaling in socially inexperienced rainbow trout larvae. This was conducted by collecting newly emerged larvae, from artificial spawning nests, and letting similar sized larvae with different yolk volumes interact in pairs. Brains were collected after 24 hours and analyzed for 5-HT and its catabolite 5-HIAA in isolated and interacting fish with big and small yolks. The ratio between 5-HT and 5-HIAA were used as an index of 5-HT activity.

## Materials and Methods

All animal procedures used in this study followed the policy and ethics as described by FELASA, Federation of European Laboratory Animal Science Associations. The Technical University of Denmark Aquaculture division holds, according to the national legislation of Denmark, the rights for fish husbandry, including tagging and humane sacrifice. DTU Aqua was given this authorization through the Ministry of Food, Agriculture and Fisheries, Danish veterinary and Food administration, Section for Aquaculture. The experiments in the present paper were conducted according to this national legislation, and the institutional guidelines at DTU Aqua. More specifically, marking of animals did not exceed the authority given to DTU Aqua nor did it exceed the Danish legislation on animal protection §10, where marking has been performed while animals were anesthetized, and no lasting suffering or limitations was inflicted to the animal. Termination of all experimental animals were conducted according to the legislation on animal protection § 12. All animals were terminated using MS 222 and no pain was inflicted.

### Experimental Protocol

The larvae used in the present study originated from commercial rainbow trout. Eggs were reared in incubator trays with a temperature varying between 7 and 10°C, at the Technical University of Denmark, DTU aqua, the North Sea Research Park, Hirtshals, Denmark. Larvae were transferred from the incubator trays to three artificial spawning nests after hatching. In short, these artificial spawning nests consisted of three parts; a flat holding tray with golf balls as bottom substrate, an emergence tunnel, and a sampling net, as described by Vaz-Serrano et al. [41]. After being placed in the artificial spawning nest, the larvae lay dormant until they reached an appropriate developmental stage and started to emerge in search of food. A water current running through the system transported the larvae into the emergence tunnel and onto the sampling nets from which they were collected every 3 h until all test subjects had emerged. In the current study the emergence period started 443 day degrees post-fertilization (DDF; the average temperature multiplied per day of incubation) and lasted for 14 days (140 DDF).

Larvae sampled from the collecting nets were immediately separated and isolated in 30 ml falcon tubes, thereafter photographs were taken and used for calculations of body and yolk size as described in Andersson et al. (2011). In short, 2-D digital images of larvae were processed using image J (National Institute of Health, USA version 1.42). The yolk was considered as a prolate spheroid, and the volume of the larval body was determined by calculating the volume of the head (sphere) and the larval body (cylinder). Remaining yolk size was determined by calculating the volume (mm^3^) of the yolk and the larval body. The remaining yolk was then determined using the following equation:
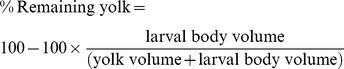



Subsequently, larvae with big yolks (10–15% of the total larvae volume) or small yolks (0–5%), were kept for further experimentation. These groups of larvae were collected during the first 4 days of emergence from the artificial nests. After this time the yolk amount was less than 10%. An analysis of covariance (ANCOVA) was conducted to investigate if the experimental groups differed in yolk size independently of body volume, and the results demonstrated a significant difference in yolk sac size between small and big in all experimental groups (groups are described more thoroughly in the following section; Round 1, F1.32 = 37.33; p<0.001; round 2, F1.17 = 209.06; p<0.001; Socially inexperienced larvae, F1.15 = 43.39; p<0.001).

After being classified as having big or small yolks larvae were marked with different colored visible implant elastomes (VIE; Northwest Marine Technology INC, Washington, USA) according to a modified method described by Jensen et al. [42]. Larvae were anesthetized in MS 222 and placed on a piece of wet paper underneath a stereo microscope. The marker was injected, using 0.3 cc insertion needles, through the skin separating the larval body from the yolk sac, making the marker visible to the naked eye. After marking, all experimental animals were left isolated in 30 ml Falcon tubes (containing 10°C water) to recover for a period of 30 min before being transferred to observation aquariums. Behavior was studied in pairs of larvae kept in opaque containers (50×60×50 mm) partly submerged in a 10 liter aquaria with circulating 10°C water, in which larvae were kept in pairs for a period of 24 h. A mesh in one side of the observation containers allowed water exchange. Each pair was visually isolated, and was video recorded for a period of 2 h directly following insertion into the observation container. Video recordings were analyzed to determine the social status of the larvae (see below).

This study consisted of two experimental rounds. In the first round, eggs and larvae from three full-sib families were used. The families were kept separated throughout the experimental round. Following emergence 5 pairs from each family were collected and allowed to interact. These pairs consisted of larvae with different yolk size (see above) but similar larval body sizes (15% within pair deviation in larval body size and no significant difference within pairs with small and big yolks; t = 0.38, P<0.71). Furthermore there were there no significant differences between total body volume between the interacting fish (t = −1.6, P<0.12). The second round of experiments was conducted to ensure that the behavioral data, from round one, were not biased by a higher total volume in the group with bigger yolks. In this round eggs and larvae originated from a mixed batch of eggs (mixed eggs from 3 females were sired by 3 males). In this round eight pairs with similar total size (15% within pair deviation and no significant difference within pairs; t = 0.053, P<0.95) but different yolk sizes (see above) were allowed to interact. Larvae had a total volume of 185±45 mm^3^ in experimental round 1 and 221±22 mm^3^ in round 2.

In experimental rounds 1 and 2 each pair were video filmed for the two first hours after being inserted in the observation chambers. After filming, the larvae were kept in the observation chambers until the next day.

### Social Dominance

The video recordings of each larval pair were analyzed to determine the social status of the larvae. Social dominance was determined by counting aggressive attacks, and dominance was considered to be established when one of the aggressive individuals became unidirectional while the other combatant only performed avoidance behavior. The aggressive acts studied in the present paper were biting, pushing and charging. Socially inexperienced larvae, consisting of 16 larvae, 8 with large yolks and 8 with small yolks, were kept isolated in the observation aquariums. These larvae originated from the same families as used in experimental round 1. Three larvae with large yolks and three larvae with small yolks originated from two of the three families, and two larvae with large yolks and two larvae with small yolks originated from the third family. Socially isolated larvae and larvae from experimental round 1 were terminated with an overdose of MS 222 after being held in the observations aquarium for 24 h. The larval brains were then rapidly dissected under a stereo microscope, frozen in liquid nitrogen and stored at −80°C for analysis of brain monamines and their metabolites.

### Assay of Brain Serotonin

The larval brains were weighed, after which they were homogenized in Sodium acetate buffer (pH = 5; consisting of 3 g sodium acetate, 0.4% glacial acetic acid (100%), 16 pellets NaOH and internal standard (Dihydroxy benzylamine hydrobromide, 98% sigma)) using an ultrasonic disintegrator. Thereafter they were centrifuged at 17,000 rpm for 5 min. The supernatant was removed and analyzed by high-performance liquid chromatography (HPLC). The monamines 5-HT, 5- Dopamine (DA), and Noradrenalin and the metabolite of 5-HT and Hydroxyindoleacetic acid (5-HIAA) were quantified using HPLC with electrochemical detection. The HPLC system consisted of a solvent-delivery system (Shimadzu, LC-10AD), an autoinjector (Famos, Spark), a reverse phase column (4.6 mm´100 mm, Hichrom, C18, 3.5 mm) and an ESA Coulochem II detector (ESA, Bedford, MA, USA) with two electrodes at −40 mV and +320 mV. A conditioning electrode with a potential of +40 mV was employed before the analytical electrodes to oxidize any contaminants. The mobile phase consisted of 86.25 mmol l^−1^ sodium phosphate, 1.4 mmol l^−1^ sodium octyl sulphate and 12.26 µmol l^−1^ EDTA in deionized (resistance 18.2 MW) water containing 7% acetonitril brought to pH 3.1 with phosphoric acid. Samples were quantified by comparison with standard solutions of known concentrations and corrected for recovery of the internal standard using HPLC software (CSW, DataApex Ltd, the Czech Republic).

In the current study we used the ratio of the catabolite to its parent monoamine as an estimation of central monoaminergic activity; 5-HT-ergic activity [5-HIAA]/[5-HT]. Because of interacting peaks, concentrations of the NE metabolite 3-Methoxy-4-hydroxyphenylglycol (MHPG), the DA metabolite 3,4-Dihydroxyphenylacetic acid (DOPAC), and NE-ergic and DA-ergic activity could not be detected.

### Statistics

All data are presented as mean ± SE, if not otherwise stated. A Pearson chi-square test was applied to determine if the frequency of individuals having big or small yolks differed between groups of larvae turning dominant or subordinate. Data on brain monoamines, their metabolites and monaminergic activity were not normally distributed, and differences between socially inexperienced larvae with big and small yolks, and socially dominant and subordinate larvae, were investigated using Mann-Whitney U-tests. Statistical analyses were conducted using Statistica (version 10.0, Stat-Soft, Inc, Tulsa, USA).

## Results

### Social Behavior

On average larvae performed the first aggressive act 31±8.9 min after being inserted in the observation chambers. The average fighting time before the aggression turned unidirectional was 9.0±1.2 min. During this time the dominant and subordinate larvae performed respectively, 5.2±1.0 and 2.5±1.0 aggressive acts. After establishment of the dominant/subordinate relationship, the dominant individuals performed 2.3±0.37 aggressive acts, while the subordinate fish did not perform any aggression during the remaining time of the 2 h observation time.

**Figure 1:**
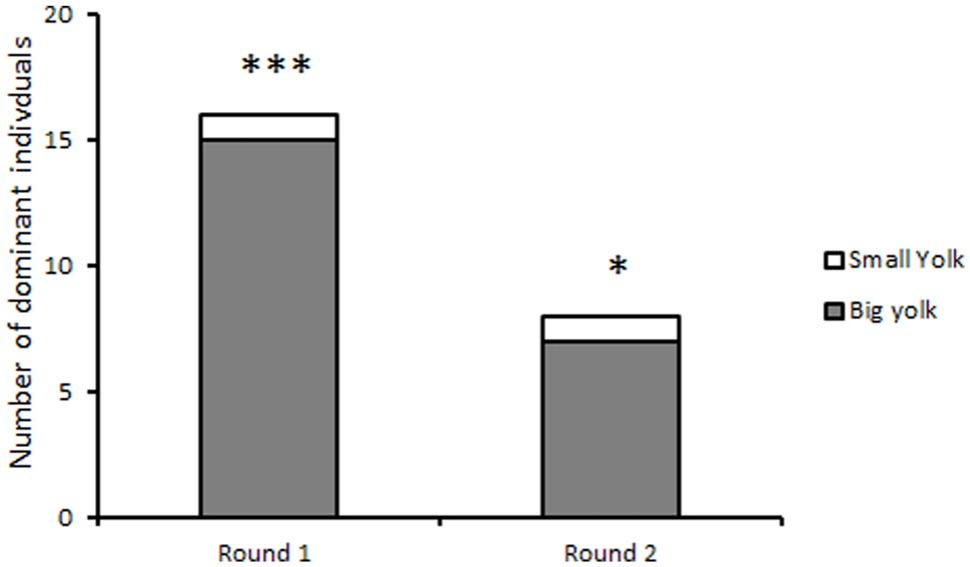
Numbers of larvae with big or small yolks that turned dominant after being allowed to interact in pairs for 24 h. In round one larvae with different yolk size but no difference in embryo body size were allowed to interact. In round two larvae with different yolk size but with similar total volume were allowed to interact. n = 15 in round one and n = 8 in round 2, *denotes P<0.05 and ***P<0.001.

In round 1 of the experiments, where larvae with different sized yolks but no difference in larval body size were allowed to interact, 14 out of 15 individuals turning dominant had big yolks (Pearson chi-square test, P<0.001) (Fig. 1). Analogous results were observed in round 2, where larvae with different sized yolks, but with similar total volume were allowed to interact; the individuals with larger yolks became dominant in 7 out of 8 pairs (Pearson chi-square test P = 0.032), Fig. 1.

### Brain Monoamines

**Figure 2:**
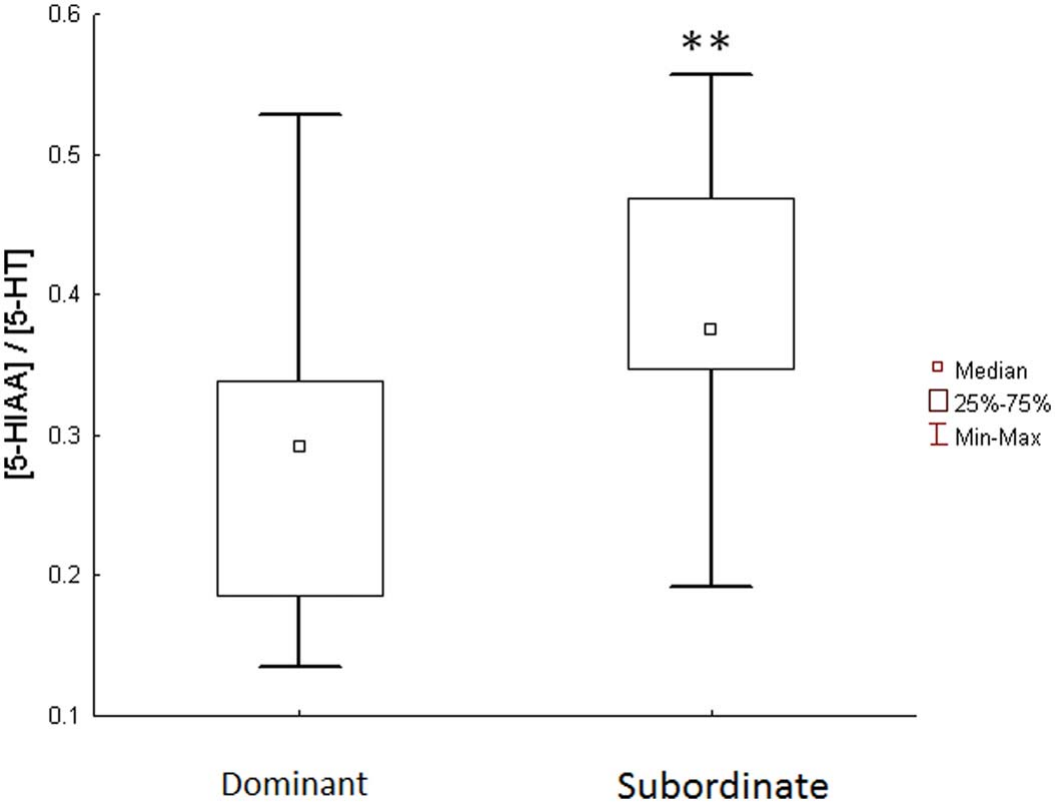
Serotonergic activity quantified as the [5-HIAA]/[5-HT] ratio in subordinate and dominant larvae after 24 h of interaction in pairs. Pairs consisted of larvae with different yolk size but no difference in embryo body size. n = 15, ** denotes P<0.005.

Socially subordinate individuals had significantly higher brain [5-HIAA]/[5-HT] ratios (Z = 2.7, P<0.005) (Fig. 2) and tended to have increased levels of 5-HIAA (Z = −1.9, P<0.057) compared to dominant individuals after 24 h of social interactions (Table 1). Apart from these effects, there were no other significant (P>0.26) effects of social interaction on brain monoamines, their catabolites or the ratio between catabolite to its parent monoamine (Table 1).

**Table 1:** Concentrations of monoamines and monoamine catabolites, and the ratio of concentrations of monoamine catoblite to parent monoamine neurotransmitter in newly emerged socially inexperienced larvae with small and big yolk.

	dominant				subordinate					
	median	25%	75%	n	median	25%	75%	n	Z	P
[5-HIAA]	170	120	210	15	229.17	193.67	243.81	15	−1.9	0.06
[5-HT]	600	560	650	15	573.76	548.28	607.45	15	0.83	0.41
[DOPAC]	7.2	6.8	14	10	6.887	4.71	9.23	12	0.89	0.37
[DA]	700	6000	820	15	660	620	730	15	0.45	0.65
[MHPG]	N.D.				N.D.					
[NE]	940	780	1332.30	14	960	900	1200	13	0.02	0.98
[5-HIAA]/[5-HT]	0.29143	0.1916	0.3246	15	0.37	0.35	0.46	15	−2.73	0.01
[DOPAC]/[DA]	0.01503	0.00911	0.0182	10	0.011	0.0071	0.015	12	1.1	0.26
[MHPG]/[NE]	N.D.				N.D					

Concentrations are ng g^−1^ brain tissue.

ND, not detectable; DA, dopamine, DOPAC, 3,4-dihydroxyphenylacetic acid; NE, norepinephrine; MHPG, 3-methoxy-4- hydroxyphenylglycol; 5-HT, serotonin; 5-HIAA, 5-hydroxyindoleacetic acid.

25 and 75% corresponds to the lower and upper quartiles respectively.

**Figure 3:**
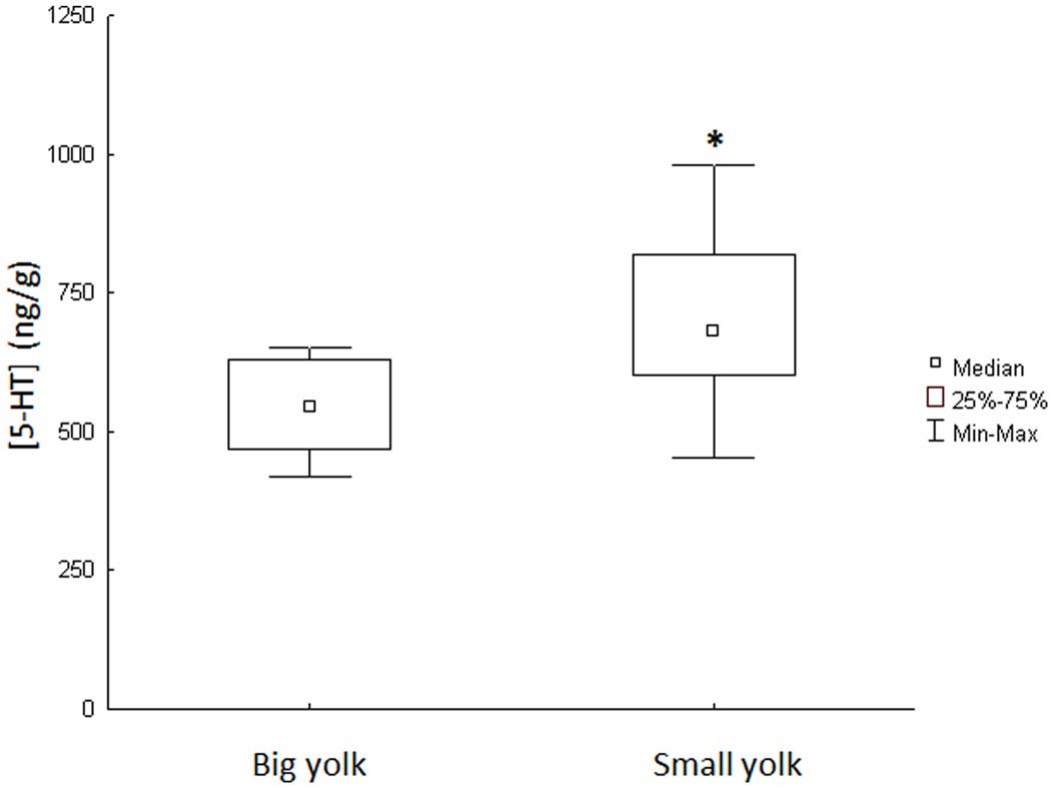
Brain concentration of 5-HT in socially inexperienced larvae with big or small yolks. n = 8, *denotes P<0.05.

In socially inexperienced larvae that were kept isolated 24 h after emergence, brain concentrations of 5-HT were significantly lower in individuals with big yolks compared to individuals with small yolks (Z = −2.1, P<0.023) (Fig. 3). This was also reflected in a trend for a higher [5-HIAA]/[5-HT] ratio in individuals with big yolks (Z = 1.8, 0.067). Apart from these effects, there were no other significant (P>0.26) differences between socially inexperienced larvae with different sized yolks on brain monoamines, their catabolites or the ratio between catabolite to its parent monoamine (Table 2).

**Table 2:** Concentrations of monoamines and monoamine catabolites, and the ratio of concentrations of monoamine catoblite to parent monoamine neurotransmitter in newly emerged socially inexperienced larvae with small and big yolk.

	big yolk				small yolk					
	median	25%	75%	n	median	25%	75%	n	Z	P
[5-HIAA]	140	130	180	8	140	130	170	8	0.052	0.98
[5-HT]	540	490	630	8	680	620	799.53	8	−2.1	0.04
[DOPAC]	5.2	2.9	8	7	2.4	1.7	11	4	0.85	0.41
[DA]	560	460	580	8	630	510	860	8	−1.2	0.22
[MHPG]	N.D.				N.D.					
[NE]	880	780	1200	8	870	950	1200	8	−0.05	0.96
[5-HIAA]/[5-HT]	0.32	0.25	0.3545	8	0.21	0.18	0.25	8	1.8	0.07
[DOPAC]/[DA]	0.0088	0.0056	0.016	7	0.0031	0.002	0.014	4	0.66	0.5
[MHPG]/[NE]	N.D.				N.D.					

Concentrations are ng g^−1^ brain tissue.

ND, not detectable; DA, dopamine, DOPAC, 3,4-dihydroxyphenylacetic acid; NE, norepinephrine; MHPG, 3-methoxy-4- hydroxyphenylglycol; 5-HT, serotonin; 5-HIAA, 5-hydroxyindoleacetic acid.

25 and 75% corresponds to the lower and upper quartiles respectively.

## Discussion

The current study demonstrates a propensity to become socially dominant in larvae that emerge from the spawning nest with large yolks. This was demonstrated by allowing larvae, with the same emergence time, to interact in pairs.

Previous studies of rainbow trout lines, selected for high and low post-stress plasma cortisol, demonstrate that these lines differ in boldness and in the propensity for social dominance (Reviewed by Øverli et al. [43]). Moreover, in a recent study it was shown that these lines specific differences were related to offspring characteristics. Females from the strain showing a more bold behavior and a propensity for social dominance produced eggs and larvae with bigger yolks [40]. In the latter study, it was hypothesized that a higher amount of unconsumed yolk could fuel a more energetically expensive aggressive and bold personality during the initial establishment of territory in newly emerged rainbow trout larvae. This hypothesis is supported by a recent study performed by Regnier et al. [44] demonstrating that individuals with the highest energetic requirements emerge with larger energy reserves. The present study not only concurs that emerging individuals vary in the amount of energy reserves, but also demonstrates a relationship between yolk reserves, aggressive behavior and the propensity to become socially dominant. Taken together, the results presented here further support the hypothesis that larger energetic reserves supply fuel for higher levels of aggression and social dominance during the initial territory establishment in newly emerged salmonid fishes.

In the current study, the relationship between yolk size and social status was present between offspring originating from the same family, demonstrating that this co-variation of aggression and energy reserves is present within sibling groups. This is consistent with other studies, demonstrating a rather high inter-family variation in personality [45]. This sibling variation has been suggested to reduce inter-family competition, by allowing siblings to capitalize on different resources [11] However, in salmonid fish there is generally a high mortality during the time of emergence and territory establishment [38], suggesting a rather limited effect of such reduced inter-family competition.

The central 5-HT system is remarkably well conserved, functionally as well as anatomically, across the vertebrate subphylum [32]. Moreover, this neurotransmitter plays an important role in integrating the behavioral and neuroendocrinal responses involved in social behavior. There is a general consensus that lower levels of 5-HT are associated with high levels of aggression [12], [46]. However, 5-HT transmission is highly sensitive to the social environment, and most studies investigate the relationship between 5-HT and aggression have been performed in individuals having previous social experiences [36], and the knowledge of whether this relationship is present in socially naive individuals is poor. The results from the present study demonstrate a higher probability of obtaining social dominance in larvae with larger yolks. Furthermore, brain levels of 5-HT in newly emerged socially inexperienced larvae was lower in individuals with larger yolks compared to individuals with small yolks. This demonstrates that a relationship between 5-HT transmission and propensity for social dominance are present prior to the initial social interaction, and that other factors apart from the social environment are causing this variation in 5-HT transmission. In humans the predisposition to aggressive behavior appears to be related to a polymorphism of genes that influence the central 5-HT levels, by affecting 5-HT production rate, synaptic release and degradation [47]. Among these variants, functional polymorphisms in the monoamine oxidase A and the 5-HT transporter have been associated with individual variation in aggression and personality in humans and in rhesus monkeys [48], [49]. However, the current experiment was not designed to reveal whether such genetic components underlie the relationship between differences in 5-HT transmission in socially naïve individuals, aggression, and yolk size at emergence. Moreover, in the present study, variation in 5-HT expression was observed between individuals originating from the same families, suggesting that an interfamily variation in 5–HT transmission is present in socially naïve salmonid larvae. Further studies are needed to investigate the mechanisms underlying interfamily variation in 5-HT transmission and its involvement in aggressive behavior in newly emerged salmonid larvae.

It has been demonstrated that the 5-HT system influence genesis, differentiation and maturation of neuron cells in certain brain regions, control of sensory inputs, stimulus processing, and motor output, during early development [48]. This neurotransmitter is also involved in the organization and development of its own neural projection pattern [50]. Furthermore, interplay between genes and environment during certain stages of neurodevelopment may lead to circumscribed brain alterations [48]. For example, such mechanisms have been demonstrated in socially isolated post-weaning rats, where long lasting alternations in behavior, brain architecture, and 5-HT neurotransmission were observed (reviewed by Fone and Porkes [29]). In the present study it was demonstrated that social subordination resulted in higher activity of the 5-HT-ergic system in rainbow trout larvae, which are consistent with the general consensus that stress elevates 5-HT activity [34]. Social experiences in association with emergence and territory establishment have been suggested to have a profound long lasting effect on behavior [41]. It is plausible that such behavioral changes are related to socially induced changes in 5-HT activity during this ontogenic niche shift. Taken together with the results presented here, which demonstrate that differences in 5-HT transmission are present in socially naive larvae, it is tempting to suggest that this neurotransmitter is involved in a gene/environment interplay during the initial establishment of a territory, and that this shapes personality in newly emerged larvae.

The results presented in the current study raise questions regarding the paternal and maternal contribution to the co-variation between aggression and yolk size at emergence. Generally, egg size is determined by the mothers genotype combined with her environmental experiences [51], [52]. Furthermore, it has been demonstrated in a study in brown trout that offspring hatching from larger eggs are also larger at the time of emergence. This suggests that much of the energy endowed by the mother to the offspring is utilized for growth and development [38]. The strong relationship between maternal influence and offspring characteristics, observed in salmonid fishes, indicates that the propensity to become dominant in larvae with large yolk reserves could be inherited from the mother. Although, in a study performed by Andersson and co-workers [40], it was demonstrated that not all energy available to the larvae was invested in growth and development, and it was suggested that this “excess” energy is related to personality traits expressed later during development. Moreover, in crickets, it has been demonstrated that there are paternal effects involved in larval size at hatch, and post-hatch metabolism [53], suggesting that male genetics contributes to how the energy is distributed within an egg. However, further studies are required to investigate the paternal link to yolk used for tissue growth and if it is related to personality differences in salmonid fishes.

In conclusion; larvae that emerge from the spawning nest with larger proportions of unconsumed yolk have a propensity to become socially dominant. A relationship which was present in siblings, suggests a link between interfamily variation in energy reserves and dominance in early emerging salmonid fish. Lower brain 5-HT levels in larvae with a propensity for social dominance is consistent with the general consensus that high levels of aggressive behavior are associated with low levels of brain 5-HT. Furthermore, that this variation in 5-HT was observed in individuals prior to the initial establishment of territories suggests that differences in aggression is mediated by a variation in 5-HT production and/or degradation, which is regulated by factors other than social environment. Social subordination during the initial defense of territory resulted in elevated 5-HT-ergic activity, which might effect the connectivity of the brain and the behavioral trait trajectories in individuals.
